# Improving Research on Transportation Interventions in Health Care

**DOI:** 10.1111/1475-6773.70122

**Published:** 2026-06

**Authors:** Na’amah Razon, Laura M. Gottlieb

**Affiliations:** 1 Department of Family and Community Medicine, University of California, Sacramento, California, USA; 2 Division of Nephrology, Department of Internal Medicine, University of California, Sacramento, California, USA; 3 Center for Healthcare Policy and Research, University of California, Sacramento, California, USA; 4 Department of Family and Community Medicine, University of California, San Francisco, California, USA; 5 Social Interventions Research and Evaluation Network, University of California, San Francisco, California, USA

## 1 | Introduction

The current issue’s article by Ianni et al. [1] examines the impacts of transportation benefits offered to low-income and disabled Medicare Advantage beneficiaries. The current paper notwithstanding, transportation benefits’ design and utilization in managed care systems have been surprisingly understudied, particularly in the context of a growing and consistent literature that documents associations between transportation insecurity and poor health outcomes across multiple conditions [2–7] and preventive care [8]. Patient-reported data suggest transportation insecurity is a common reason for both missed appointments and delayed care [9]. In short, no one would suggest that transportation insecurity is itself *good* for health.

Given transportation insecurity’s generally negative influence on health, multiple strategies have emerged to address beneficiaries’ transportation needs in different healthcare environments. Though traditional Medicare plans have offered only limited transportation benefits, in 2019, CMS expanded the definition of “primarily health related” and thereby enabled Medicare Advantage (MA) plans to include supplemental benefits to better address members’ transportation needs [10, 11]. The result has been a near 14% expansion in transportation offerings across MA plans, and plans in counties with high transportation barriers are especially likely to provide nonemergency medical transportation (NEMT) benefits [12].

In light of the recent expansion of NEMT benefits, we read with great interest the Ianni, et al. article, which examines the impact of offering transportation benefits on care utilization among low income and disabled MA beneficiaries. Like the authors, we were surprised by the study’s largely null results: “We detect no evidence of meaningful change in E&M [Evaluation & Management], procedure, imaging, annual wellness, ambulance or ER care among low income and disabled beneficiaries in plans offering NEMT in 2019.” Since transportation insecurity is both common among MA beneficiaries [13] and associated with increase in emergency department and hospitalization [14], why did this study find no healthcare utilization impacts from the benefits designed to improve it?

As Ianni and colleagues acknowledge, the main challenge in interpreting the study’s findings is the gap between offering benefits and subsequent use of these services. The new work expertly examines the available MA benefit data. Yet translating the authors’ findings to inform policy decisions requires appreciating the underlying reasons why offering NEMT services did not lead to expected healthcare utilization changes, and only then developing relevant policy solutions. Below we outline three possible explanations for this gap.

### 1.1 | Beneficiaries Did Not Need the Benefits

The most immediate possibility is that beneficiaries simply did not need the newly offered MA NEMT benefits. The analysis focused on low income (dual Medicare and Medicaid members and those qualifying for a Low Income Subsidy) and those eligible for MA based on a disability. These groups may already have access to alternative transportation benefits and therefore may not benefit from additional transportation services offered by MA plans. For example, Medicaid requires plans to offer beneficiaries transportation services to medically necessary services and Paratransit services are available to people living with eligible disabilities. Despite the intuitive appeal of the theory that beneficiaries already have access to alternative transportation services and therefore do not need the MA transportation benefit, existing data do not support this hypothesis. Evidence suggests many people with MA are indeed transportation insecure [15, 16], perhaps because NEMT and Paratransit services are themselves difficult to navigate. Medicaid NEMT use is consistently low (4%–5%) across the United States and Paratransit services are not available to individuals living in areas with public transportation deficits [17, 18].

### 1.2 | Lack of Awareness About Benefits

An alternative possibility is that members were just not aware of MA’s new NEMT benefits, especially during the first year of these services when Ianni and colleagues examined benefits uptake. Though no research has specifically studied MA members’ awareness of transportation benefits, research examining the underuse of vision and dental benefits in MA suggests that nearly half of MA enrollees were not aware that they had dental or vision coverage [19]. In a strategy specifically designed to address the general lack of awareness about supplemental benefits, a 2024 CMS rule required that starting in 2025, MA plans engage in tailored outreach efforts to members so that enrollees would be aware of the supplemental benefits available to them, including a list of any supplemental benefits not accessed by the member during the first 6 months of the year and instructions about how to access the benefit. The rule was removed under the Trump administration, so no federal regulation requires plans to address gaps in awareness about supplemental benefits coverage [20].

### 1.3 | Benefit Design

Perhaps the most underappreciated possibility to explain Ianni, et al.’s findings is that the services being offered did not meet members’ underlying transportation needs. Despite increased prevalence of some NEMT benefits across plans, these benefits are not standardized, meaning plans differ in the number of rides covered, allowable distances, and advanced notification periods. Members also have reported poor quality and reliability of services; members with disabilities, low literacy, or low technology capability indicate that while transportation benefits are critical for mobility and health, rides arrive late, no show, or are incompatible with their mobility needs (e.g., cannot accommodate wheelchairs) [21, 22]. Ultimately, uncertainty about availability and adequacy is likely to influence transportation services use.

## 2 | Next Up: Developing Patient-Oriented Transportation Solutions

The upshot is that comprehensive transportation solutions will require evaluations not only about benefits design but also about benefits uptake, and ideally, on the comparative use of different transportation programs. The good news is that the transportation sector has already started testing client-centered solutions that might be adopted in MA—solutions such as mobility wallet and basic universal mobility interventions [23, 24]—and may be more likely to shape members’ health and utilization outcomes. These approaches provide users more flexibility, for example, enabling users to pay for gas, taxis, or ride shares, or other ride options.

While health services researchers, clinicians, and beneficiaries advocate for those innovations to reach MA, strategies to improve transportation benefits in the shorter term can include increasing member education and outreach about benefits, standardizing advanced notice requirements, establishing reasonable pick-up windows, developing clear grievance processes, and holding transportation vendors accountable for high quality services. Combined, these changes are likely to increase consumers’ knowledge, confidence, and use of MA NEMT transportation services.

## Data Availability

Data sharing not applicable to this article as no datasets were generated or analysed during the current study.
